# “Going into the black box”: a policy analysis of how the World Health Organization uses evidence to inform guideline recommendations

**DOI:** 10.3389/fpubh.2024.1292475

**Published:** 2024-03-22

**Authors:** Heather Ingold, Gabriela B. Gomez, David Stuckler, Anna Vassall, Mitzy Gafos

**Affiliations:** ^1^Department of Global Health and Development, Faculty of Public Health Policy, London School of Hygiene and Tropical Medicine, London, United Kingdom; ^2^Unitaid, Global Health Campus, Geneva, Switzerland; ^3^Department of Social Sciences and Politics, Bocconi University, Milan, Italy

**Keywords:** public health guidelines, evidence-to-decision framework, complex interventions, qualitative research, stakeholder engagement, expert opinion

## Abstract

**Background:**

The World Health Organization (WHO) plays a crucial role in producing global guidelines. In response to previous criticism, WHO has made efforts to enhance the process of guideline development, aiming for greater systematicity and transparency. However, it remains unclear whether these changes have effectively addressed these earlier critiques. This paper examines the policy process employed by WHO to inform guideline recommendations, using the update of the WHO Consolidated HIV Testing Services (HTS) Guidelines as a case study.

**Methods:**

We observed guideline development meetings and conducted semi-structured interviews with key participants involved in the WHO guideline-making process. The interviews were recorded, transcribed, and analysed thematically. The data were deductively coded and analysed in line with the main themes from a published conceptual framework for context-based evidence-based decision making: introduction, interpretation, and application of evidence.

**Results:**

The HTS guideline update was characterized by an inclusive and transparent process, involving a wide range of stakeholders. However, it was noted that not all stakeholders could participate equally due to gaps in training and preparation, particularly regarding the complexity of the Grading Recommendations Assessment Development Evaluation (GRADE) framework. We also found that WHO does not set priorities for which or how many guidelines should be produced each year and does not systematically evaluate the implementation of their recommendations. Our interviews revealed disconnects in the evidence synthesis process, starting from the development of systematic review protocols. While GRADE prioritizes evidence from RCTs, the Guideline Development Group (GDG) heavily emphasized “other” GRADE domains for which little or no evidence was available from the systematic reviews. As a result, expert judgements and opinions played a role in making recommendations. Finally, the role of donors and their presence as observers during GDG meetings was not clearly defined.

**Conclusion:**

We found a need for a different approach to evidence synthesis due to the diverse range of global guidelines produced by WHO. Ideally, the evidence synthesis should be broad enough to capture evidence from different types of studies for all domains in the GRADE framework. Greater structure is required in formulating GDGs and clarifying the role of donors through the process.

## Background

The World Health Organization (WHO) plays a crucial role in developing global guidelines that inform public health policies and practices worldwide. In the years 2020–2021, WHO developed over 290 guidelines (1), making it a prominent policymaker, particularly during the COVID-19 pandemic. However, this increased visibility has also brought greater scrutiny to the organization and its processes, raising concerns about the transparency of the process and evidence base of the guidelines being produced.

**Figure 1:**
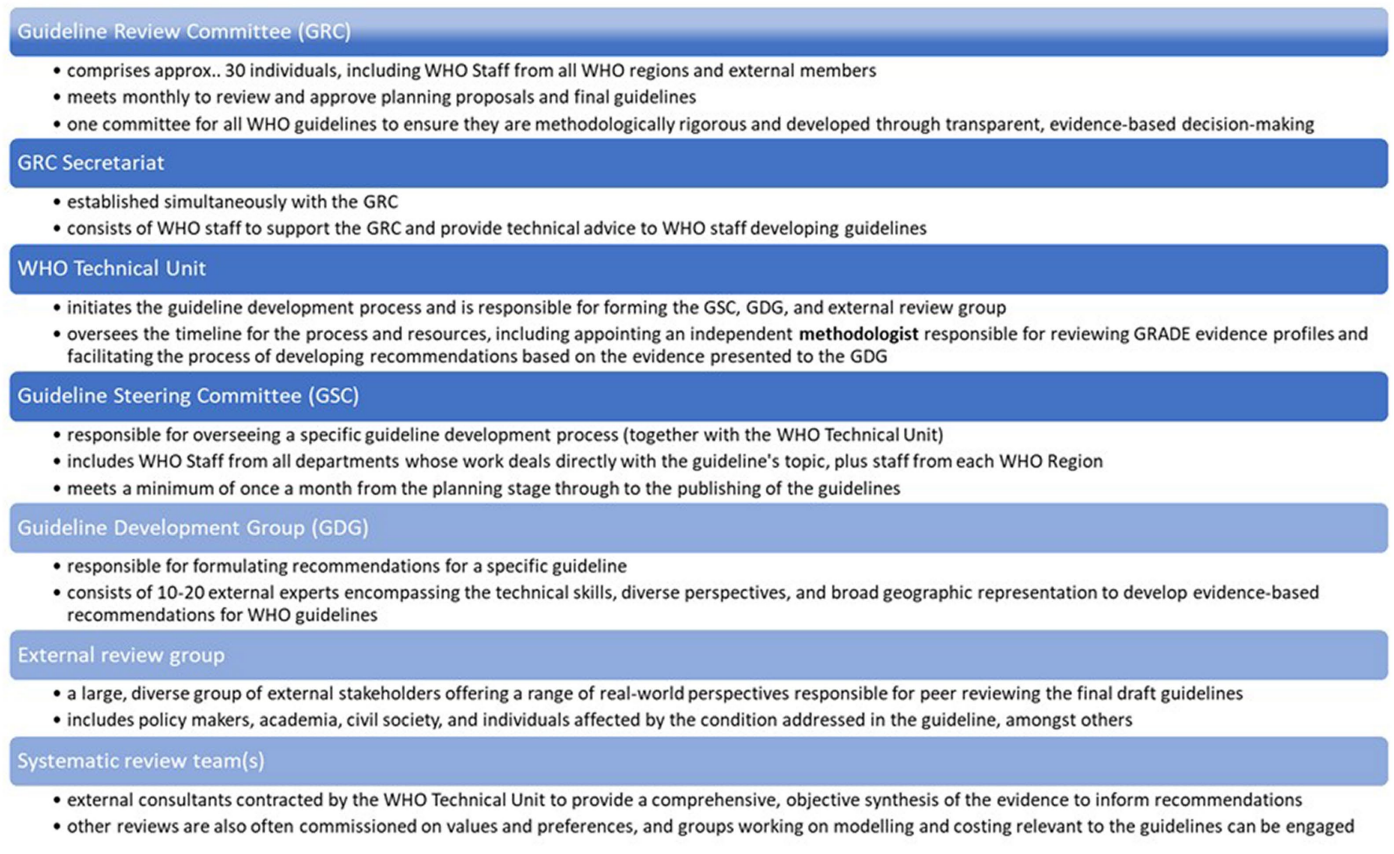
Main contributors to the WHO guidelines process.

WHO aims to produce high-quality guidelines that directly contribute to measurable improvements in people’s health at the country level (2). In 2006, WHO faced criticism for not adhering to its own “Guidelines for Guidelines,” as recommendations were found to be based on expert opinion rather than systematic evidence-based methods (2, 3). In response, WHO established the Guideline Review Committee (GRC) to ensure that its guidelines adhere to high methodological standards and are developed through a transparent, evidence-based decision-making process (4). Since the establishment of the GRC in 2007, WHO has been committed to improving the guideline development process, emphasizing systematic and transparent approaches (5–7). The GRC convenes monthly to review guideline planning proposals and final draft guidelines, ensuring compliance with WHO standards before publication. Various key groups are involved in the development of WHO guidelines, as illustrated in Figure 1. The guideline process consists of three stages: planning, development, and publishing/dissemination, following the Grading Recommendations Assessment Development Evaluation (GRADE) framework (8, 9).

In mid-2017, WHO initiated a comprehensive transformation effort to reposition, reconfigure, and re-capacitate the Organization. As part of this transformation, a Science Division was established, which centralizes the creation of norms and standards, and is led by the first Chief Scientist appointed in March 2019.

It is unclear whether these changes have addressed the previous criticisms of WHO’s guideline process. Previous studies have faced challenges in independently evaluating the WHO guideline-making process due to restricted access to its internal workings. Independent researchers have been limited to partial insights, such as, observations of external reviews of GRC-approved WHO guidelines (5) or external reviews focusing on the quality of WHO-developed guidelines (6, 10). The most recent independent evaluation of WHO’s entire norms and standards function was published in 2017 (11). This evaluation looked at 10 global public health goods, including two guidelines, and followed them from initiation and design to dissemination and incorporation at country level. The aim was to explore if, how and why these products contributed towards fulfilling WHO’s normative function; not to assess the technical content. It found that WHO needed a succinct definition of normative products and functions, lacked a corporate plan to define and prioritize normative products, that more than half of the products were initiated internally by WHO staff, and that the extent to which WHO publications reach their targeted audiences was mixed. While these studies have identified weaknesses in the guidelines themselves, they have not been able to delve into the “black box” of WHO’s guideline process to examine if the development process contributes to these weaknesses.

This study aims to explore the policy process through which WHO utilizes evidence to inform guideline recommendations, using the update of the WHO Consolidated HIV Testing Services (HTS) Guidelines in 2019 as a case study. The study objectives are as follows: (1) analyse WHO’s guideline process, including the use of evidence to inform recommendations; (2) evaluate the involvement of key stakeholders, including expert selection and engagement, management of conflicts of interest, and stakeholder interactions with each other and the evidence; and (3) identify major internal and external “tensions” influencing the process. We chose the word “tension” to refer the disconnect between a goal or desire and what happens.

## Methods

The conceptual approach of this study is guided by a framework developed by Dobrow et al. (12) for expert groups involved in health policy recommendation development. Recognizing the shift from evidence-based medicine, where decisions are regarding the care of individual patients, to evidence-based policy, where decisions shift to the population level, Dobrow et al. looked at the evidence supporting two distinct orientations to what constituted evidence: the philosophical-normative and the practical-operational. The former focuses on the quality of evidence and evidence hierarchies, while the latter focuses more on relevance, applicability, and generalizability to a specific context. The framework has three main stages: the introduction, interpretation, and application of evidence, which are underpinned by internal and external contextual factors.

**Figure 2:**
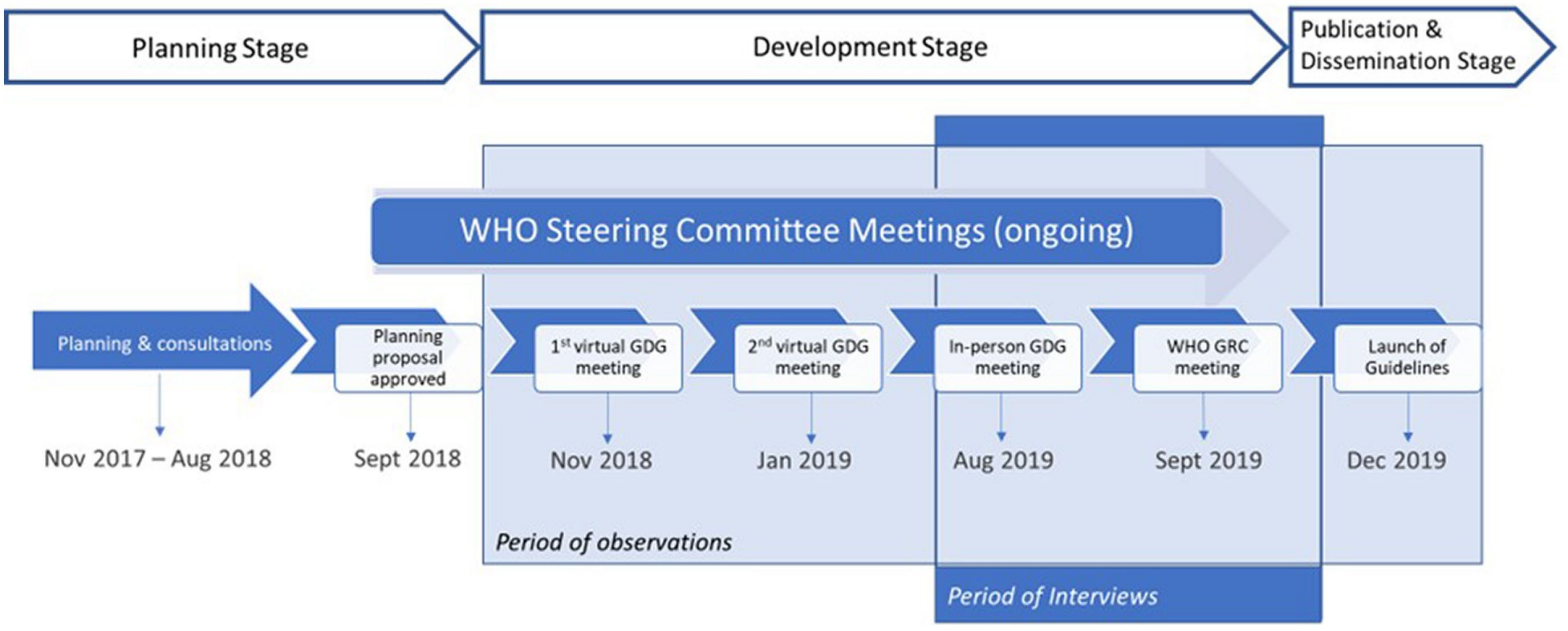
Timeline and milestones for the WHO consolidated HTS guideline update.

We observed a series of guideline development meetings and performed semi-structured interviews with key participants in the WHO guideline-making process. WHO’s guideline process has been described in detail elsewhere (7); Figure 2 highlights the specific process for updating the Consolidated HTS guidelines on which this case study is based, and the key points in the process where we observed meetings. We also conducted a desk-based review of 14 guidance related documents corresponding to the major steps of WHO’s guideline process (Appendix 1). The first author reviewed all documents and used the handbook to better understand the process and inform the interview guide before commencing the study. The documents related to the Consolidated HTS guidelines were received and reviewed throughout the case study to better understand interactions and discussions across the various stakeholders, and how these related to decisions made using the evidence for this guideline update.

### Meeting observation

A total of 16 meetings were observed for this case study, including GRC, Guideline Steering Committee (GSC), and Guideline Development Group (GDG) meetings (Table 1). We developed an observation guide (Appendix 2) referring to the approach developed by Kawulich (13) outlining participant observation as a data collection method to ensure a systematic recording of important elements, with the flexibility to allow the focus to organically change based on emerging themes (14).

**Table 1:** Virtual and in-person meeting observations.

Meetings observed	Virtual	In-person
WHO Guideline Steering Committee	12	—
Guideline Development Group	2	1
WHO Guideline Review Committee	—	1

As part of these observations, we observed the 2 days face-to-face GDG meeting in Geneva, Switzerland in August 2019. It is a critical meeting for WHO’s guideline process because it’s when the GDG reviews the quality of evidence and decides on the direction and strength of each recommendation using the GRADE framework. We also observed the GRC meeting in September 2019, where the final guideline was discussed and approved for dissemination. Observing these two critical meetings allowed us to better understand how key stakeholders interacted with each other and with the evidence materials during two important decision-making processes.

### Semi structured interviews

We commenced interviews from August 2019, after the systematic reviews were completed and the in-person GDG decision-making meeting occurred to understand how the evidence was used to inform recommendations.

We recruited participants for interview by obtaining a list of names for each key stakeholder group involved in the guideline update (Figure 1) and used the randomization function in Excel to select individuals from each of the key groups (Table 2). While there was the possibility to identify additional informants if data saturation was not achieved, it was not necessary because the response rate was 100%.

**Table 2:** Semi-structured interview participants (***n*** = 13).

Participant category	WHO	Gov’t	CSO	Academia	Other	Total
WHO Technical Team	3					3
WHO Guideline Steering Committee	1					1
WHO Guideline Review Committee	1					1
Guideline Development Group		1	3	1		5
Methodologist					1	1
Systematic Review Team(s)				2		2
Total	4	1	3	3	1	13

We conducted 13 semi-structured interviews in English, five in-person and seven over the phone, all lasting between 45–90 min. A semi-structured interview guide was developed with themes related to WHO’s guideline process, stakeholder involvement and engagement, guideline scoping, and the use of evidence to inform recommendations (Appendix 3).

All interviews were audio-recorded and detailed field notes were taken during and after each interview to incorporate into the analysis. We transcribed the audio recordings using Otter.ai. and performed quality control by listening to the audio file and reading the transcription for accuracy. All transcriptions and field notes were deidentified and each interviewee was assigned a unique code. Due to the number of interviews conducted, we are purposefully not including characteristics of individuals after quotations used in this paper to avoid identification.

### Data analysis and interpretation

We coded interview data using the qualitative data analysis software Quirkos. Each interview transcription was uploaded into Quirkos and then manually coded through the software. Fieldnotes from meeting observations and reflections and contextual information about interviews were included in the coding.

We followed the recommendations of Braun and Clarke (15), to identify main themes in qualitative interviews and our analysis followed Bazeley’s (16) three stage approach of reading, reflecting, and coding; describing and comparing; and refining. In the first pass, we interpreted the data inductively without reference to a framework with the intention of allowing themes to emerge from the data. To address study objectives 1 and 2, we examined emerging themes, existing literature, and our understanding of WHO’s guideline process from the desk-based review and iteratively updated our coding framework.

**Figure 3:**
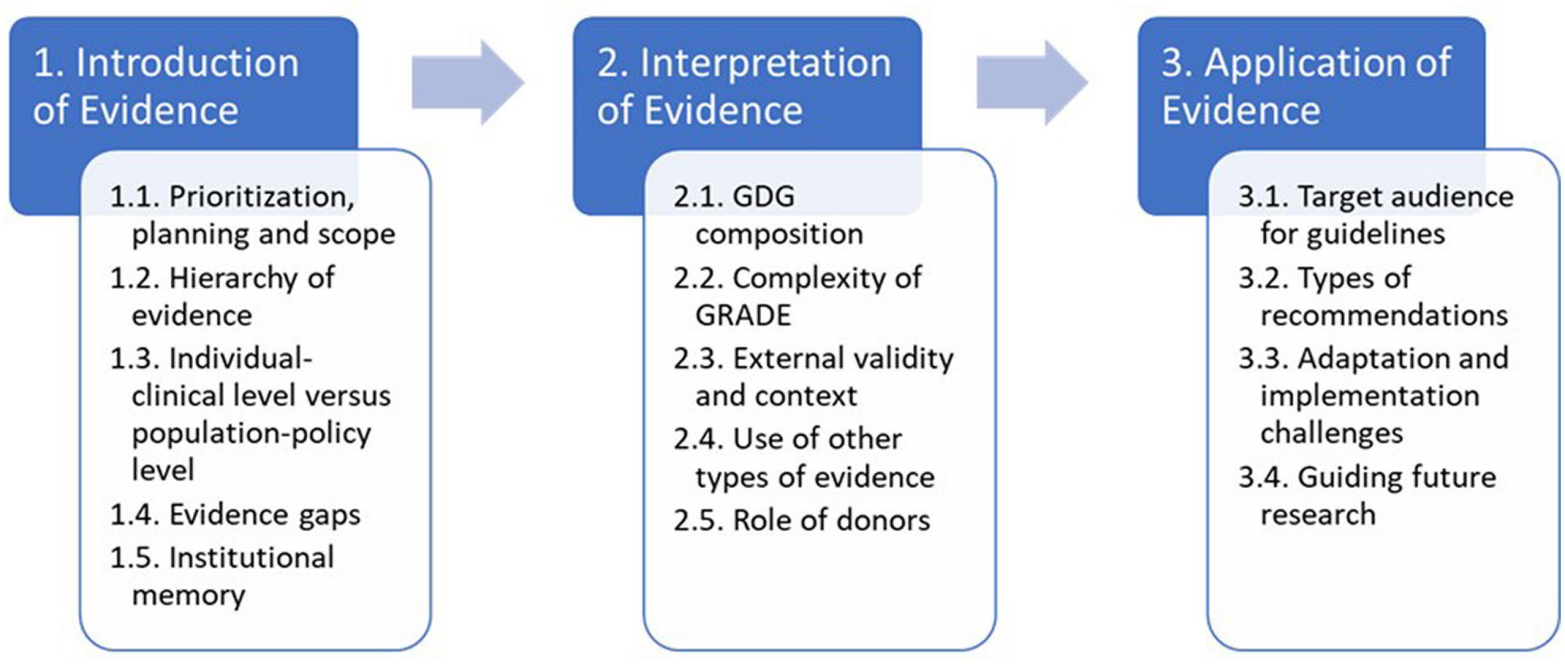
Summary of results through the lends of a context-based evidence-based decision-making framework.

Then in the second pass, a deductive step, we mapped codes to the three main stages from the Dubrow et al. conceptual framework. After analysing the clusters, we merged codes into 3–5 main themes for each stage. To address study objective 3, we took a final inductive step to integrate and analyse our data to identify a series of “tensions” in WHO’s guideline-development process. We created a thematic mapping of these according to the Dobrow et al. framework (Figure 3).

Ethics approval was obtained through the LSHTM Ethics Committee in July 2018 (Ref. # 15321). Before the interviews, all participants received an English language study information sheet and provided written informed consent (Appendix 4). Participation was not reimbursed or incentivised. Data were anonymised using participant codes and were stored electronically in secure data files on a password-protected and encrypted laptop.

## Results

This section presents the main findings of the case study according to the three main stages of the Dobrow et al. framework: introduction, interpretation, and application of evidence. We have interwoven the findings related to internal and external factors throughout the three main stages: including key stakeholders and their impacts on evidence-based decision making. Figure 3 provides a summary of the main tensions and serves as a guide for the presentation of the findings.

### Stage 1 introduction of evidence

We identified potential tensions related to prioritization, planning and scope; hierarchy of evidence; individual-clinical level versus population-policy level; and evidence gaps.

#### Prioritization, planning, and scope

In general, we found no organizational-level prioritization of guidelines or global/regional consultation of needs. Guidelines would often appear without warning to in-country stakeholders. This lack of coordination and communication was noted by a WHO staff member: “*WHO now has this global good process to prioritize what key questions we need to be focusing on. But there’s not any screening of whether a topic is a priority. If you look at the list of guidelines we put out, the extent to which they are organized or prioritized on a sense of global public goods is not happening*.”

The process for the update of the consolidated HTS guidelines was well-planned and organized, with early involvement of the methodologist. As noted by one of the interviewees: “*What was done particularly well for this guideline, is preparation in advance so that things were not done at the last minute or on the fly. It’s highly unusual, often WHO is acting in a more outbreak control mentality, and research needs not to be an emergency*.” What also seemed to differ was early input on the scope of the guidelines from a wide variety of stakeholders from different geographies and genders including Ministries of Health (MoHs), donors, technical partners, civil society, people living with HIV, and other key stakeholders. Such a wide consultation and high level of inclusiveness was appreciated by almost all interviewed and was echoed by the GRC during the meeting we observed in September 2019.

Turning to the guideline scope, our analysis found that the systematic review protocols appeared to neglect important information relevant to the GDG decision on the direction and strength of a recommendation. To make a final decision required data not just about efficacy but also about values and preferences, acceptability, feasibility, equity, and resource use. However, the protocols were designed to focus narrowly on efficacy: “*There were the quantitative outcomes, and then there were the values and preferences and the cost and feasibility sections. I was surprised how little emphasis they were given during the protocol development process. They heavily weighed on the GDG but were the least developed areas of the review and the least rigorously done*.”

#### Hierarchy of evidence

Half of the interviewees suggested that GRADE’s emphasis on a hierarchy of evidence focuses too narrowly on randomised control trials (RCTs), even when they might not be the appropriate study design. There were four systematic reviews for this guideline update focused on efficacy (does it work); albeit some PICOs (Population, Intervention, Comparator, and Outcome) were worded with a focus on effectiveness (does it work in the real world). Following the GRADE framework, RCT evidence should be prioritized, but if no RCTs are found, the next study design in the hierarchy of evidence should be used (17, 18). This is regardless of the type of question being asked. In this case, the four systematic reviews were broad ranging from “*should* social network-based approaches be used as an additional approach to HTS” and “*should* HIV self-testing be used as an additional approach to HTS” to “which demand creation strategies are effective for HTS” and “*should* western blotting be used within HIV testing algorithms”: two found RCTs and did not look further; one used a so-called “living meta-analyses” from the hivst.org website, which updates in real-time; and the fourth did not find RCTs and needed to search further, including non-randomized studies (NRS). The focus on RCT evidence led to some of the interviewees questioning the definition of evidence. “*What is considered evidence is in the first place the issue. Because WHO was so nervous from the time we were criticized, that some of the guidelines were based on expert opinion, that we have gone totally in the other direction*.”

Another challenge raised was that systematic reviews used a narrowly focused RCT search filter. Half of the interviewees mentioned this tension, asking, “are you finding everything that is out there?” “*One review used the Cochrane sensitive and precise RCT string, but it dramatically cuts down the results. A lot was missing for values and preference, and costs related to the demand creation review. It was the weakest part of the review*.”

#### Individual-clinical level versus population-policy level

Throughout the process, we observed a tension between the reliance on individual-clinical level data and the need to inform population-wide policy. One interviewee noted that RCTs might work well to answer individual questions but may not be adequate for capturing real world effectiveness at the population level. “*Sometimes we need to be very clear that we have individuals and what the outcomes are for them. And then we have programmes and what the outcomes are for those, which might not be best answered in the RCT setting*.”

Another suggested that RCT evidence may, in fact, not be useful at all: “*The quantitative data will tell you the* ‘*what,*’ *the qualitative data will be able to focus much more on the* ‘*how*’ *and the* ‘*why.*’ *So, when you start looking at information around acceptability, or uptake, I find RCT evidence is pretty useless*.” Curiously, several interviewees pointed out that, despite the reliance on individual-clinical data, the scope of the guideline update was in as much clinical as it was population-wide, and implementation focused.

#### Evidence gaps

During the in-person GDG meeting, we observed several GDG members noting the lack of evidence from certain WHO regions, such as Latin America and Asia. Over two-thirds of the interviewees acknowledged the lack of evidence outside of high-income countries and the Sub-Saharan Africa region for this guideline update. All expressed the importance of having evidence from other regions, especially regions with different contexts, legislation, epidemics, political will, and other external factors: “*It’s the state of science in HIV, we have this polarity that is not always representative (of) what is happening all over the world*.”

Another potential weakness is related to the representativeness of the evidence in terms of the populations it should serve. Two GDG members, one WHO staff, and two academics interviewed asked, “if RCTs are based on small, unrepresentative population groups, how can evidence from those RCTs be used for global guidance?” “*If we continue to exclude women from RCTs or key populations, that it puts those who are responsible for reviewing and making judgments about the evidence in a difficult position to take a conservative view and to say that the evidence only applies to men and not to women. And not even considering pregnant women. In the demand creation review, there were plenty of RCTs, but we are not talking about a clinical intervention*.”

A key part of finalizing the scope of the systematic review is the GDG’s ranking of the outcomes for each PICO question. Two academics interviewed noted that the GDG ranked outcomes as a high priority for which the systematic reviews found little or no evidence. Whereas there was evidence for some of the non-priority outcomes. However, in practice only those prioritised outcomes are presented during GDG discussions, so this effectively excluded available evidence on “non-prioritized” outcomes from decision-making. “*The GDG prioritized a bunch of things, which I knew at the beginning that there was no evidence on, and we were unlikely to have evidence on. But they ended up being quite important, and then we had to say there was no evidence. How does WHO handle it when evidence exists in an important area, but the GDG is not interested in it?*” Linked with the above finding that the systematic review protocols did not include evidence for acceptability, feasibility, equity and resource use, the net effect was that, ultimately, some decisions were made based on expert opinion.

### Stage 2 interpretation of evidence

In this stage, we uncovered five areas of tension including the composition of the GDG; complexity of GRADE; external validity and context; the use of other types of evidence; and the role of donors.

#### GDG composition

While the establishment of the GRC and the use of GRADE aimed to ensure evidence-based recommendations, interviewees believed that recommendations relied on expert opinion due to the lack of evidence for most of the “other” GRADE domains: values and preferences, balance of benefits and harms, resource implications, priority of the problem, equity and human rights, acceptability, and feasibility. One interviewee indicated that two GDGs looking at the same evidence could come up with a different recommendation. This further supported the belief of all interviewees that context and the diverse experience of the GDG members is just as important as the evidence. “*Different people brought a lot of concerns about contextual relevance that I would not have thought of, and I felt like the process was very inclusive and gave as much time honestly in the decision-making process to those contextual considerations as they did the quantitative evidence from the literature*.”

Concerns were raised about the selection process for GDG members with varying approaches reported, including choosing “friends” or “naysayers” to influence recommendations. GDGs are considered non-expert advisory groups to WHO and despite having a formal guideline process, there is no formal, transparent process for selecting GDG members. Because the HTS guideline was an update to an existing guideline, about half of the GDG members were from the 2015 group and the WHO Technical Unit consulted with WHO Regional and Country offices for new members resulting in a diverse group. One WHO staff explained for this guideline update: “*We do not ask our friends. We do not ask people because we think they’ll be easy. I’ve heard people in other guidelines groups saying that he’s very difficult and will not invite him. He can be very controversial. It’s better to involve people who might oppose what you want because they are more likely to understand why you are making that decision if they are part of the process*.” The diverse experience and contextual considerations of GDG members, especially real-world, country-level experience, were seen as important in the decision-making process, especially for “other” GRADE domains.

#### Complexity of GRADE

GDG members interviewed appreciated the transparency and process of GRADE, but found it highly complex and requiring significant self-teaching and learning. To our knowledge, WHO does not provide holistic training for external stakeholders, including academics conducting systematic reviews who are new to GRADE. As one GDG member noted, “*We had some sort of crash training. I did my reading, but you also learn as you go through the process*.”

The GRADE tables, which display the quantitative data from the systematic review and are used to determine the certainty of evidence, were seen as long, difficult to read, and not readily accessible to non-specialized audiences. This raised questions about how GDG members without extensive training in GRADE could make decisions on the quality of evidence. An interviewee noted that it is very easy for things to “get lost” in the GRADE tables: “*They are not easy to read. They just end up with so many footnotes, and it’s hard to get. It may be useful if you are writing something, but when you are interpreting it, it is a lot harder to read and understand*.”

#### External validity and context

The tension between internal validity prioritized by academics, and the focus of GRADE, and external validity prioritized by GDG members was noted. Context and understanding the applicability of evidence in different environments were considered crucial by GDG members during decision making: “*I think that even the people who are in the GDG have less buy in to the strength of the evidence because it excluded context and the types of evidence that they rely on*.”

The Evidence to Decision (EtD) table, which contains the factors required to determine the direction and strength of a recommendation, often lacked evidence for “other” GRADE domains beyond the quality of evidence. Yet, what came out strongly in the interviews as well as in our observation of the in-person GDG meeting is that these “other” domains were almost more important during the decision making because they represented evidence in real-world settings: “*My sense is it’s not the EtD table that’s influencing the decision of the strength of recommendation. It is the evidence of benefits and harms, the other domains like equity and resource use, the desire and the will, and the expertise and the knowledge of the people on the GDG that say, well, this is what the evidence tells us now, but we still believe that this needs to be a strong recommendation*.”

#### Use of other types of evidence

The issue of prioritisation of RCT evidence in the introduction of evidence stage also surfaced in relation to this stage. Over half of the interviewees highlighted the importance of including observational studies, and reflected that qualitative evidence could have been helpful, for example, with the systematic review looking at social network-based approaches being used as an additional approach to HTS. Knowing that something works in a controlled setting is important in clinical settings, but knowing why something works, for whom and in what situations seemed to be important for public health recommendations. Context was one of the key themes during the in-person GDG meeting.

Two-thirds of those interviewed advocated for the need to find a better way to link research evidence with programme data. One interviewee stated the importance of considering implementation outcomes because it is more of what MoHs and decision-makers want to know: “*I do think that in some situations, GRADE can be quite limiting. And that, there may be situations where we do not need any more RCTs in HIV Testing*.”

Cost, cost-effectiveness, and affordability were other areas where evidence was lacking but weighed heavily on the GDG during the decision-making process. While costing data was found for some of the systematic reviews, it was recognized that they were not as robust as they could have been. Generalizing economic data from an individual country or region is complex and understanding the cost and cost effectiveness of an intervention is important but going beyond this to a budget impact analysis is critical for national-level decision-making. The GRC highlighted the importance of including economic information more formally in WHO’s guideline process in its meeting we observed when they approved the final guideline. As one interviewee noted: “*WHO has a chief scientist, but they also need a chief economist*.”

#### Role of donors

We uncovered an area of disagreement regarding the role of donors in WHO’s guideline process. Donors in this context refer to the funder of the specific guideline or a funder of HIV programmes, not a donor or Member State contributing funding more broadly to WHO. WHO’s handbook for guideline development states that donors of a guideline cannot play a role in the guideline process and should not influence the recommendations (7). It further notes that private funders, like foundations, may observe GDG meetings but cannot contribute in any way, and that if government agencies [e.g., United States Agency for International Development (USAID)] fund the guidelines their employees may not sit on the GDG. However, it also states that there can be exceptions when a person is considered essential due to their expertise in relation to the guideline.

For the HTS guideline update, the WHO Technical Unit involved donors in the scoping stage, they attended the in-person GDG meeting as observers, took part in the external peer review, and were engaged in disseminating and implementing the final guideline. GDG members interviewed all had positive views about including donors as observers to the GDG meeting: “*It’s important that you identify the financers of the countries and then make them available in the observational group or as stakeholders because they are one of the key players in most of the countries*.”

However, it was mixed for WHO staff interviewed. Three had similar views as the GDG members, while two noted that formally the donors of the guidelines should not have a role, even as observers: “*Donors are not allowed to be part of the GDG. There are grey areas in terms of whether they can be observers or not. Currently, there are no red lines in having donors being observers at meetings. However, it is becoming a bit fuzzy. For example, say a state-funded institution is funding a guideline, and you have a representative from that institution in the GDG. Increasingly, the GRC is becoming a little bit more against it because the line is a little bit too thin. So, it’s a little bit of an area of uncertainty we do not have a strict application of, but maybe we need to err on the side of caution*.”

It is important to note that the guideline process we studied took place prior to COVID-19 and the final GDG meeting was in person. Donors could interact with GDG members directly during coffee breaks, dinners, and outside the meeting rooms. We did not observe any donor attempting to steer the process or influence GDG members. Since COVID-19, WHO has held all GDG meetings virtually, which may limit these concerns.

### Stage 3 application of evidence

It is important to note that while our study concluded once the final guideline update was approved by the GRC in September 2019, the final guideline was not published until December 2019 and the scope of this study did not encompass dissemination or implementation. However, we identified four areas of tension in this stage: the target audience for guidelines; the types of recommendations; adaptation and implementation challenges; and prioritizing future research.

#### Target audience for guidelines

A key question raised in the interviews was who is the end user of the guideline: policy makers, clinicians, programme managers? This is not surprising because it is also not clear in the final guideline approved by the GRC, which broadly states “*Countries and other end-users*.” These end users have very different needs making it impossible to capture them all in a single document. Adding to the mix are the different WHO stakeholders, including the GRC, who must approve the final guidelines: “*The way that GRC makes you report guidelines is very technical. You put in a huge amount of effort to get this polished document to GRC, which has every bit of the GRADE process all done beautifully. And then you have got a very unwieldy document, which no one can really use. I do not know how you could do it differently*.”

Interestingly, this was also acknowledged by the GRC themselves during their meeting in September 2019 when the final guideline was approved: “*The guideline provides clear cross-referencing to other related WHO guideline standards and although the document is substantial—it is well presented and clear, although seems to serve multiple purposes, and derivative documents will be helpful in pulling out specific aspects of the document tailored to different audiences*.”

#### Types of recommendations

We found that a strong recommendation was perceived as better because it is more likely to be included in national programmes. Those interviewed that had been through WHO’s guideline process before noted that there is sometimes a reluctance at WHO to have conditional recommendations because countries may not adopt it, and donors may not pay for it. This leads to what GRADE defines as discordant recommendations when the quality of evidence is low, but the GDG makes a strong recommendation. WHO has many discordant recommendations (19–25).

However, the updated HTS guideline had context-specific recommendations for HIV self-testing and social network approaches to testing. This was appreciated by the GRC and its Secretariat as well as one GDG members interviewed. Unfortunately, it was noted that this is not consistent across WHO: “*Conditional recommendations are meant to allow for context, but there’s this real reluctance to have conditional recommendations. Although this HIV guideline does not even have conditional recommendations. It’s got context-specific recommendations. I think it’s brilliant for global guidelines to go into that level, and we should see more of this in WHO recommendations*.”

#### Adaptation and implementation challenges

All GDG members interviewed stated that countries cannot keep up with the number of WHO guidelines produced, even within a single disease. Countries need time to adapt current guidelines to accommodate new recommendations. But often, by the time this is done and implemented, there is already another WHO guideline out. This flood of guidelines is linked to the lack of prioritization at WHO described in the introduction of evidence stage, but it also hints to the lack of evaluation of the outcomes from guideline recommendations, which should feed into WHO recommendations. As one interviewee noted: “*Guidelines are being updated in TB five or six times recently. HIV, I cannot even begin to tell you how many. People at the country level see us, and they run. They’re like, please do not do this to me!*”

Another point that came out strongly, is that whether a country adopts a WHO recommendation depends on many aspects, not just the evidence. Political will, availability of resources, capacity of the health system, and the state of the disease epidemic are all factors. “*WHO guidelines may not be applicable for all countries. Some countries may pick it up. Others may not. It depends on the funding scenario in the country too. It is not something they must do*.”

#### Guiding future research

There was consensus amongst all interviewed that WHO needs to improve follow up after guidelines are disseminated. Implementation outcomes from guideline recommendations are not evaluated systematically, and currently do not formally feedback into the guideline process. As one interviewee said: “*Implementation science is not taken on board enough in the guideline process. The whole implementation end of the process is the most neglected part. It does not feedback enough into guidelines*.”

Half of the interviewees said that the guideline process should be more structured with evidence gaps driving targeted research. To date, WHO guidelines do not include a separate section for evidence gaps, even though there was a session dedicated to this. The final version of the guidelines does, however, note opportunities for further research throughout.

## Discussion

Our analysis of the 2019 WHO HTS guideline update provided several insights. First, the HTS guideline update was characterized by an inclusive and transparent process, involving a wide range of stakeholders. However, it was noted that not all stakeholders, including systematic reviewers, could participate equally due to gaps in training and preparation, particularly regarding the complexity of GRADE. Additionally, the volume of information was overwhelming for most GDG members to comprehend within the given timelines. Second, we found that WHO’s GPW does not set priorities for which or how many guidelines should be produced each year. Furthermore, WHO does not systematically evaluate the implementation of their recommendations to demonstrate that evidence utilization is leading to better health outcomes, with implementation outcomes feeding back into the scope of future guidelines. Third, our interviews revealed significant disconnects in the evidence synthesis process, starting from the development of systematic review protocols. While GRADE prioritizes evidence from RCTs, the GDG heavily emphasized “other” GRADE domains for which little or no evidence was available from the systematic reviews. As a result, expert judgements and opinions played a significant role in making recommendations as observed during the in-person GDG meeting and through interviews. In fact, WHO often has what GRADE considers to be “discordant” recommendations whereby the evidence is considered “low quality,” but the GDG nonetheless makes a strong recommendation (19–25). Finally, disagreements arose regarding the presence of donors as observers during in-person GDG meetings.

### Study limitations

Before interpreting the study’s findings, it is important to acknowledge several limitations. Firstly, this analysis focuses on one guideline and may not be representative of all guideline processes at WHO. However, some tensions identified in this study were expressed as common by interviewees who had participated in multiple WHO guideline processes, suggesting that the recommendations derived from this study could enhance WHO’s guideline development. Secondly, this study examined the revision of an existing guideline, and decision making for new guidelines may differ due to a less established and more contested evidence base. Further research is required to explore similar tensions in the development of new guidelines. Thirdly, this study did not evaluate the publication and dissemination stage, warranting future research to assess whether the guideline achieved its objectives in terms of adoption by WHO member states and benefits to the end users. Fourthly, structural and policy changes within WHO were initiated during the study period. For example, WHO created a new Science Division, where the Norms and Standards Unit is housed. There are ongoing internal reviews of how expert panels are formed, to rectify some of the known tensions in how GDGs are used for guideline development. This highlights WHO’s recognition of the need for improvements, and the timeliness of the findings from this study to inform enhancements to future guideline processes. Fifthly, our interviews did not include a donor of this guideline update because they were not randomly selected. Since the study exposed a tension about the role of donors, in hindsight, purposefully selecting a donor to include their perspective could have enhanced our findings. Lastly, the qualitative interviews conducted in this study may have been influenced by social desirability bias, and it is impossible to measure if that was exacerbated or reduced by the lead investigator being a WHO staff member. However, following best practices, steps were taken to minimize potential bias and ensure the accuracy of interpretation of our findings (16, 26).

Despite these limitations, this study possesses several strengths. The lead investigator is a WHO staff member and had access to internal documents and meetings that were not accessible externally. Furthermore, the study captured the entire process from the planning proposal to final approval of the revised guideline, providing a comprehensive understanding. Additionally, the study employed a well-established theoretical framework by Dobrow et al. to analyse the desk-review, meeting observation, and qualitative interview data.

Our findings corroborate recent observations about tensions in WHO’s guideline process. Specifically, we found evidence of tensions coherent with those identified in the external evaluation of WHO’s entire norms and standards published in 2017 (6, 11). For example, several interviewees pointed to problems associated with lack of prioritization in developing guidelines, leading to a proliferation of guidelines. However, our research identified additional tensions in the process, all of which have yet to be addressed, as detailed below.

### Interpretation of the findings and literature context

After an in-depth analysis, the study findings were categorized into five tensions within the guideline-making process: prioritizing and defining the scope of guidelines; public health versus clinical guidelines; composition of the GDG; lack of clarity about the guideline’s target audience; and the role of donors.

#### Tension 1. Prioritizing and defining the scope of guidelines

One significant issue we identified is the absence of a prioritization process within WHO’s GPW to determine which guidelines should be developed and how many each year. Without centralized “top-down” prioritization or dedicated funding for guideline development, WHO Technical Units often receive requests and direct funding from donors for new/revised guidelines. This leads to a large number of published guidelines each year, essentially arising from a “bottom-up” approach. An example of this challenge is seen in maternal and perinatal health (27), where WHO has issued over 400 recommendations in the past decade, making it difficult to ensure that these recommendations remain up to date.

We also found no evidence of WHO evaluating the impact of their recommendations. Our observations, interviews, and existing literature (28) underscore the need for robust evaluation mechanisms that demonstrate how the utilization of evidence leads to improved health outcomes. Implementation outcomes should inform the scope of future guidelines to ensure that real-world evidence informs guideline recommendations. The independent evaluation also suggested that WHO should shift attention and resources from guideline preparation to the implementation process, including dissemination, adoption, adaptation to facilitate feedback and learning for future updates (11). Moreover, WHO should establish a structured, cyclical process to identify evidence gaps that drive targeted research (29–31).

With the establishment of the new Science Division at WHO Headquarters, which centralizes the creation of norms and standards, there is an opportunity to set high-level priorities across the organization. Based on the findings of this study, it may be helpful for WHO to undertake a comprehensive review of their guidelines with the aim of consolidating them into more holistic and comprehensive guidelines. In parallel, some WHO Technical Units have begun implementing “living guidelines,” that are continuously updated in real-time. While these efforts aim to respond more rapidly to emerging evidence, our study highlights the importance of considering the composition of the GDG in these “living” guidelines. The use of a “living” GDG, where membership may be long-term or undefined, could potentially exacerbate existing tensions and should be reviewed and evaluated. Further research is needed to assess the impact of “living guidelines,” including their effectiveness in facilitating more efficient and effective adoption of changes by countries.

#### Tension 2. Public health versus clinical guidelines

Our study suggests that a one-size-fits all approach to guideline development may be outdated, especially for public health guidelines. We found a need to tailor evidence synthesis to address the central question(s) of the guideline. The GRADE framework prioritised RCTs and clinical evidence hierarchies, excluding population-level evidence which could more appropriately address the guideline question and provide evidence for the “other” GRADE domains. Ideally, the evidence synthesis should be broad enough to capture evidence from different types of studies for all domains in the GRADE framework. Further evidence of a disconnect between GRADE and evidence synthesis practice could be seen in the fact that 55.5% of WHO recommendations were discordant between 2007–2012, and of these, 84.4% did not meet GRADE’s criteria for when strong recommendations should be permitted even when the underlying evidence is low (19–25). Perversely, the lack of strong evidence to answer the guideline’s originating question resulted in high weighting of expert opinion and judgements, rather than empirical evidence, contradicting WHO’s guideline principle that the evidence used to develop WHO guidelines is publicly available (7).

The situation becomes further complicated when GDG members have a poor understanding of GRADE (22). It has been widely published that GRADE was created for clinical guidelines and not for complex, public health guidelines. If GRADE could better fit public health needs, it is possible that WHO would make fewer discordant recommendations, which may also address the perception that conditional recommendations may not be adopted (25).

Due to these constraints, WHO Technical Units have modified and adapted GRADE in various ways (32–38). It may be necessary to better adapt GRADE to evolving population health needs, a process which has been underway since 2016 (39). A recent systematic review provided a mapping and feature summary of the current available approaches and tools to develop, report and assess clinical practice guidelines (40). Furthermore, Petticrew et al. (41) suggest using tools like a conceptual framework or diagram to display the inter-relationships within the wider system, portraying hypotheses about the process involved, to generate specific research questions. This may help ensure the right criteria are identified up front, allowing for a more robust evidence synthesis, avoiding situations where there is little, or no evidence found for all domains in the grading framework, especially for areas critical for the GDG in making recommendations and for countries to adopt them. Other institutions, such as the National Institute for Health and Care Excellence (NICE) in the United Kingdom, similarly recognized this tension. In 2015, NICE decided to include more robust methodologies allowing reviewers to take a broader view of evidence, using a range of search methods, and putting out calls for evidence where this was needed (42, 43). The Guidelines International Network also acknowledges the importance of appraising and including knowledge from a wide variety of sources to keep guideline development innovative and diverse (44, 45).

#### Tension 3. Composition of the GDG

Consistent with prior research (44, 46–50), we found that the GDG composition is one of the most important aspects in the guideline process because their central task is to develop evidence-based recommendations (7). Since a GDG should be comprised of a wide variety of stakeholders, what each member considers “evidence” is very different, and what the end user of the guideline considers evidence may not match either. The GDG for this guideline update was diverse with members from various regions and organizations with a lens on gender and included populations affected by HIV. However, as noted previously, the application of GRADE requires judgement and expert opinions expressed by GDG members forms an essential part of the decision-making process. Yet, WHO considers GDGs to be non-expert advisory groups and does not have formally prescribed rules of procedure in place to form these committees. WHO should look to find a balance between changing membership too frequently and using the same experts again and again by establishing a more formal procedure for GDGs.

#### Tension 4. Lack of clarity about the guideline’s target audience

A common question raised throughout this study by participants was, “who is the guideline targeting?” GRADE suggests GDGs must decide what perspective they are taking—the clinician, the policy maker, the programme manager—before commencing the process (17, 48). This is because when the GDG ranks the outcomes, the perspective of those who are affected should be used. For clinical guidelines, normally the target audiences are clinicians, thus the perspective would generally be that of the patient. However, for public health guidelines it is less clear, and the focus of policy makers are often more political and financial-focused. Further complicating the issue is a recognition that the way WHO requires guidelines to be published is very clinical-focused, requiring the creation of various supporting documents targeting different end users.

Our study suggests that a one-size-fits all approach may be outdated, especially for public health guidelines. A recent commentary announced the introduction of WHO “SMART” guidelines: Standards-based, Machine-readable, Adaptive, Requirements-based, and Testable (51). Authors suggest this new WHO-supported approach will help countries more rapidly, and effectively implement WHO guideline recommendations. However, this approach requires countries to have robust digital systems in place to receive the information electronically and strong health systems to disseminate information to regional and local levels. More research is needed as WHO begins to implement this approach.

#### Tension 5. The role of donors

The final area of contention was the role of donors in WHO’s guideline process since they can have indirect influence on WHO’s guideline process, during the planning stage or through the external peer review. For this guideline, there was lack of agreement about whether donors should be allowed to observe the in-person GDG meeting, and whether as observers, they could influence GDG recommendations since they could engage during coffee breaks or before/after the meeting commenced which were important moments of decision-making. This may have had more prominence in our study as funding for the HIV epidemic is very donor driven and the GDG meeting for decision making occurred in-person (pre-COVID-19). However, this finding is consistent with a recent study citing almost one-third of WHO guidelines did not provide information on the funder of the guideline, and of those that did report funding sources, less than half described the exact role of funders in the process (52). In the future, WHO should establish an organization-level position on the participation of donors throughout the guideline process.

In conclusion, WHO has undergone considerable change over the past few years with the creation of the Science Division, where the oversight of norms and standards has been centralized. The increased oversight, dedicated resources, and openness to change, presents an opportunity to improve the guideline development process. As such, the operational recommendations and identified research gaps from this case study come at a favourable time. This study contributes to the growing literature on the complex landscape of guideline development processes. Three key messages arose. First, WHO would benefit from a more holistic prioritization of the development of their guidelines with evaluations of their impact. Second, there is a need for a wider evidence synthesis lens due to the varying types of guidelines WHO produces. Third, more structure around the formulation of GDGs and the role of donors throughout the process is needed. While this case study focused on WHO, the findings are potentially relevant to other stakeholders who engage in the development of guidelines.

## Data availability statement

The raw data supporting the conclusions of this article will be made available by the authors, without undue reservation.

## Ethics statement

The studies involving humans were approved by London School of Hygiene and Tropical Medicine. The studies were conducted in accordance with the local legislation and institutional requirements. The participants provided their written informed consent to participate in this study.

## Author contributions

HI: Conceptualization, Formal analysis, Investigation, Writing – original draft, Writing – review & editing. GG: Conceptualization, Supervision, Writing – review & editing. DS: Supervision, Writing – review & editing. AV: Conceptualization, Supervision, Writing – review & editing. MG: Conceptualization, Methodology, Supervision, Writing – review & editing.
