# Supplementary material for: “Going into the black box”: a policy analysis of how the World Health Organization uses evidence to inform guideline recommendations
Source: Front Public Health. 2024 Mar 22;12:1292475. doi: 10.3389/fpubh.2024.1292475 (PMC10995388; doi:10.3389/fpubh.2024.1292475)
Supplement: Supplementary file 1 [file Data_Sheet_1.docx]

# Appendices

## Appendix 1: List of documents from desk-based review

| # | Documents Reviewed | Publication Date | Internal / External | Stage of Guideline Process | Description |
| --- | --- | --- | --- | --- | --- |
| 1 | [WHO handbook for guideline development*,* 2^nd^ edition](https://apps.who.int/iris/bitstream/handle/10665/145714/9789241548960_eng.pdf?sequence=1&isAllowed=y)  *(Chapters 1-13)* | 2014 | External | Preliminary | Provides an overview of the processes and methods by which a WHO guideline should be developed and the standards by which the guideline will be evaluated by the WHO GRC |
| 2 | WHO handbook for guideline development*,* 2^nd^ edition  [Chapter 14: Strong recommendations when the evidence is low quality](https://apps.who.int/iris/bitstream/handle/10665/145714/9789241548960_chap14_eng.pdf?sequence=5&isAllowed=y) | 2016 | External | Preliminary | Provides guidance to WHO staff who develop guidelines to help GDGs formulate recommendations whose strength is consistent with the level of uncertainty surrounding the underlying evidence and thus are more consistent with GRADE |
| 3 | WHO handbook for guideline development*,* 2^nd^ edition  [Chapter 15: Using evidence from qualitative research](https://apps.who.int/iris/bitstream/handle/10665/145714/9789241548960_chap15_eng.pdf?sequence=6&isAllowed=y) | 2016 | External | Preliminary | Provides guidance related to how WHO can incorporate evidence from qualitative research into guideline development and implementation to complement evidence on the effectiveness and harms of interventions and resource use |
| 4 | WHO handbook for guideline development*,* 2^nd^ edition  [Chapter 16: Decision-making for guideline development](https://apps.who.int/iris/bitstream/handle/10665/145714/9789241548960_chap16_eng.pdf?sequence=7&isAllowed=y) | 2016 | External | Preliminary | Focuses on group decision-making, primarily as conducted by GDGs at WHO |
| 5 | WHO handbook for guideline development*,* 2^nd^ edition  [Chapter 17: Developing guideline recommendations for tests and diagnostic tools](https://apps.who.int/iris/bitstream/handle/10665/145714/9789241548960-chap17-eng.pdf?sequence=20&isAllowed=y) | 2019 | External | Preliminary | Addresses how to develop guideline recommendations for tests from accuracy studies when direct evidence about the test’s effect on patient-important outcomes is lacking |
| 6 | WHO handbook for guideline development*,* 2^nd^ edition  [Chapter 18: Complexity and guidelines](https://apps.who.int/iris/bitstream/handle/10665/145714/9789241548960-chap18-eng.pdf?sequence=49&isAllowed=y) | 2019 | External | Preliminary | It aims to demonstrate the value of considering a complexity perspective in WHO guidelines and describes when and how to address complexity when developing WHO guidelines |
| 7 | Guidance on Types of Evidence for Decision Making in Guidelines | March 16, 2019 | Internal | Preliminary | Guidance for the WHO GRC on the criteria for the use of evidence to inform recommendations in WHO guidelines |
| 8 | GRADE Primer: The Why, What and How of GRADE tables: Five Steps to Understanding | April 21, 2016 | Internal | Preliminary | Created by WHO Technical Unit (HIV Testing team) as a training guide for the GDG on how to assess the quality of research evidence using the GRADE framework |
| 9 | 2019 WHO Consolidated Guidelines on HIV Testing Services – Guideline Review Committee Scoping Document | September 3, 2018 | Internal | Scoping | Planning proposal reviewed and approved by GRC |
| 10 | WHO GRC Meeting notes | September 19, 2018 | Internal | Scoping | Meeting notes from the GRC meeting for the review and approval of the planning proposal |
| 11 | WHO GSC Notes | Ongoing from August 2018 | Internal | Development | Ad hoc bi-weekly meeting notes |
| 12 | Materials for GDG meeting (in-person meeting) | August 2019 | Internal | Development | All information that the GDG is required to review and grade the evidence; incl. GRADE tables, published literature, and evidence to decision tables |
| 13 | WHO GRC Meeting Notes | September 18, 2019 | Internal | Development | Meeting notes from the GRC meeting for the review and approval of the final guideline |
| 14 | [WHO Consolidated Guidelines on HIV Testing Services](https://apps.who.int/iris/rest/bitstreams/1313903/retrieve) | December 2019 | External | Launch | Final approved updated guideline |

*Note: Internal documents were made available to WHO Staff involved (all) and to the GDG (8 & 12)*

## Appendix 2: Observation Guide

**Observation Guide**

**Study Title:** A policy analysis of how the World Health Organization (WHO) uses evidence to inform guidelines at headquarters

**Lead Investigator:** Heather Ingold

**Date: Event Observed:**

**Location (layout of room?):**

**Meeting objectives:**

**Attendees (list name, role & org):**

**Discussions related to non-clinical evidence:**

**Decisions taken:**

**Notes of non-verbal behaviours:**

**Critical reflection notes of overall event observed:**

## Appendix 3: Semi-Structured Interview Guide

**Interview Guide**

*This interview guide will be adapted after discussions with key stakeholders, and as needed as interviews proceed. The questions have been designed to gather information required to address the study objectives, based on the conceptual framework described in the protocol.*

**Opening questions**

1. Can you please tell me about your current role at your organization?
2. What is your role in this WHO guideline development process?
3. Have you participated in the WHO guideline development process before? *If yes:*
   1. *In how many WHO guideline developments have you participated,*
   2. *Which guideline(s), and*
   3. *What was your role (e.g., GSG, GDG, etc.) for each?*

**Guideline Development Process**

1. Can you walk me through the necessary steps for the WHO guideline development process? (*Probe for any pre-process steps as well as the general process; any differences across diseases or between clinical and non-clinical interventions*)
2. What is the role of the Guideline Review Committee? How are members chosen/selected? How often do they serve on the GRC?
3. What is the role of the Guideline Steering Group? Do you know how it is formed /members selected? How is this done? (*Probe for any criteria for selecting the GSG*)
4. What is the role of the Guideline Development Group? Do you know how it is formed / members are selected? How is this done. (*Probe for any criteria for selecting the GDG, including how the Chair and Vice-Chair are selected, are there any limits to serving on GDGs for the same guidelines or in general*)
5. Is this process the same for updates to existing WHO guidelines (as is the case for the WHO HIV Testing Guidelines)?

**Stakeholder Groups**

1. Who are the key stakeholders involved in the WHO guideline development process?
2. How are they selected?
3. Who selects the members for each group? (*Probe for How often is there turnover and how easily can others join a group (e.g., GSG, GDG, etc.)?)*
4. For each stakeholder mentioned, ask:
   1. *Tell me about [X] stakeholders?*
   2. *What is their role?*
   3. *What kinds of institutions do they represent (e.g., for academia)?*
5. What role do donors, including foundations, play in the development of WHO guidelines (e.g., BMGF, USAID, GF, etc.)?
6. What role do other UN organizations play in the development of WHO guidelines (e.g., UNAIDS, UNICEF, etc.)?
7. What role does industry play in the development of WHO guidelines (e.g., manufacturers, pharma)?
8. What role do members of the population that the guidelines are targeting play in the development of the WHO guidelines (e.g., PLHIV, KPs, etc.)
9. From your perspective, are there any stakeholders whom you think should be a part of the guideline development process but who are not?
10. How often does the group you belong to meet during the development/update of a guideline?
11. Do you think it was enough?
12. Could you describe your experience in working with such a diverse group of stakeholders spanning across different sectors during the WHO guideline development process?
13. How does WHO handle declaration of interests (DOI) for its GDG members?
14. Have there been any conflicts of interest that needed to be addressed?
15. If so, how were these managed throughout the WHO guideline development process?
16. Generally speaking, and since there are so many different types of stakeholders involved in this process, in what areas do they commonly agree and/or disagree?

**Guideline Scoping and Use of Evidence**

1. The WHO Handbook for Guideline Development notes that resolving uncertainty should be the focus of most WHO guidelines. What does *resolving uncertainty* mean to you?
2. Do you know how priorities are set when WHO decides to update guidelines? If so:
   1. *Can you describe the process for setting these priorities?*
   2. *Who is involved in this process?*
   3. *Who is responsible for taking a final decision on the priorities?*
3. Do you know how the scope of the guidelines is determined? If so:
   1. How are the background questions determined? (*Probe for the process, who is involved in the process and who is responsible for taking a final decision*)
   2. How are the key questions formulated (PICO)? (*Probe for the process, who is involved in the process and who is responsible for taking a final decision*)
   3. How are the outcomes determined and prioritized? (*Probe for the process, who is involved in the process and who is responsible for taking a final decision*)
4. Do you think there were any key questions – or important outcomes – that were not included? If so, why?
5. Once the key questions are set, can you describe the process to determine the study designs included in the systematic reviews?
   1. *What do you think of this process (is it effective, is it appropriate)?*
   2. *How do you think this process could be improved?*
   3. *Probe to see if the WHO department decides or if the GSC/GDG is involved; are they are tailored to the type of intervention; how non-clinical interventions are handled as well as location of evidence (HIC versus LMIC)*
6. Do you have experience using the Grading of Recommendations Assessment, Development and Evaluation (GRADE) to assess evidence?
   1. If so, can you tell me about what that experience was like?
   2. *Probe on the types of evidence used, was it useful, was it easy to understand, was it appropriate and useful for the type of guideline produced*
7. Do you have experience using GRADE or a GRADE-equivalent assessment process for qualitative / non-randomized evidence (like GRADE CERQual or ROBINS-I) or for complex interventions (like WHO-INTEGRATE evidence to decision framework)?
   1. If so, can you tell me about what that experience was like?
   2. *Probe for how qualitative evidence or evidence from NRS was used*
8. Apart from the systematic review evidence looked at through GRADE what other evidence was considered in this WHO guideline development process or others you were involved in?
   1. *Probe for how information from the background questions were used as well as if values & preferences, cost, cost-effectiveness, feasibility, resource implications, risk-benefit, or even programme data/experience were considered*
9. In your experience how were these ‘other types’ of evidence used / weighed? (*Probe for how they were incorporated into GRADE tables and/or in the final decision making or not*)
10. How much time was devoted to reviewing evidence versus other tasks?
11. What kind of evidence do you value most – and why?
12. What kind of evidence do you value least – and why?
13. What kind of stakeholders tended to advocate for which kinds of evidence?
14. How is their input received from the group as a whole?
15. WHO guidelines are usually made on a global basis. How well do you think regional, national, or sub-national variations are addressed or included in recommendations?
    1. *Are these variations discussed during the process? If so, how often (elaborate)?*
    2. *How are they integrated into decision making / recommendations?*
    3. *Do you think that all regions are appropriately represented in the various groups (e.g., GSC or GDG) to help bring perspective from global to regional/country level?*
    4. *Are their voices heard?*
    5. *How could the process be improved to improve adoption at country level?*

**Closing**

1. In your experience, what are some of the challenges in developing global guidelines for WHO?
2. What have you experienced that has worked well?
3. How would you improve the guideline development process to optimize the use of evidence and resources?
   1. *Probe if they could change anything about the WHO guideline development process, what would it be – e.g., human resources, use of GRADE, use of other ways of assessing non-clinical evidence, time, etc.?*
4. There is still a debate about using the same grading system for clinical and public health (or health system) interventions, including at WHO. *What is your current view on this?*
5. Is there any other relevant information that you may want to add?
6. Can you recommend other stakeholders to be interviewed?

**Thank you!**

## Appendix 4: Participant Consent Form

**Title of Project:** A policy analysis of how the World Health Organization (WHO) uses evidence to inform guidelines at headquarters

**Name of PI/Researcher responsible for project:** Heather Ingold**,** DrPH candidate at LSHTM

| **Statement** | **Please initial each box** |
| --- | --- |
| I confirm that I have read the information sheet dated…………………. (version…….) for the above-named study. I have had the opportunity to consider the information, ask questions and have these answered satisfactorily. |  |
| I understand that my participation is voluntary and that I am free to withdraw at any time without giving any reason. |  |
| I understand that the investigator will not use any specific quotations from this interview in any report or paper without my explicit permission.  I understand that the investigator will contact me to seek said permission and that I am free to decline the use of my quotations in any report or paper. I understand that by use of my quotations in any report or paper will be anonymous and not attributable to me. |  |
| I agree to take part in the above-named study and for my interview to be recorded. |  |

|  |  |  |
| --- | --- | --- |

Printed name of participant Signature of participant Date
